# An integrating contextual approach using architectural procedural modeling and augmented reality in residential buildings: the case of Amman city

**DOI:** 10.1016/j.heliyon.2022.e10040

**Published:** 2022-08-11

**Authors:** Maha AlFadalat, Wael Al-Azhari

**Affiliations:** The University of Jordan, Department of Architecture Engineering, Amman, Jordan

**Keywords:** Contextual design theory, Procedural modeling, Generative design, Shape grammar, Augmented reality

## Abstract

In architecture, context term refers to the surrounding area in which the building is situated. Context is crucial in architectural theory and practice when developing architectural vocabulary. Creating harmony with historic context amidst expanding development has been a primary concern and focus of designers since the mid-twentieth century. Contextualism theory refers to the interaction between new buildings and their surroundings, as well as the challenge of integrating new and existing structures to establish congruence and continuity. When using a contextual design method, an analysis of traditional architectural style has a considerable impact on a designer’s decision-making process (Lambe and Dongre, 2019). In this study, a general framework is proposed, which integrates shape grammar-based procedural modeling and augmented reality technology to create a harmonious environment through the generation of new designs based on the grammar of the existing architectural style of the residential buildings in Amman city without limiting the designer’s creativity of order to address the issue of Western architectural movements influencing the architectural style in Amman, which is incompatible with the city’s identity and it’s context. This approach was tested on a group of University of Jordan students, and the results were evaluated using a machine learning model. The effectiveness of this method was discovered, and it can be regarded as a step toward achieving spatial congruence.

## 1 Introduction

Architecture is described as "a true reflection of society," created at a certain time for a specific location and people. These elements define the built environment’s location, conditions, and surroundings; in other words, they provide context for the built form. Society has been responding to such situations to produce better living environments since the advent of civilization and the emergence of protected areas as shelters. Structures erected at various periods of evolution are tangible manifestations of people’s aspirations and provide a sense of ‘identity’ to a place (Lambe and Dongre, 2019).

Responding to this identity through new development designs has always been a big challenge for architectural practices. It has been observed that new development constantly emphasizes universal appeal while losing architectural character and continuity. A discordant environment is created by haphazard and unsympathetic new development as in-fill in a traditional location. ‘Responding to the situation’ is frequently suggested as a solution to the problem. Contextualism has been addressed using a variety of methodologies and theories, all of these methods necessitate a thorough grasp of the surrounding environment. In order to achieve the genius loci of the site, a contextual design approach would be desirable (Lambe and Dongre, 2019).

In this study, procedural modeling approach is suggested as a methodological approach to address the issue of contextual design. The residential buildings in the city of Amman were studied in the context of their traditional value. The study explored shape grammar and the most important common features of residential buildings in the city of Amman as a tool to analyze existing design in the generation of new designs in cooperation with augmented reality technology, thus creating a harmonious environment.

## 2 Related work

The relevant resources for our approach were briefly discussed in the preceding paragraph; more specifics will be provided in the following topics.

### 2.1 Contextual design

In architecture, contextual design is an approach of achieving a harmonious architectural environment, where new structures must be compatible with their environment (Liauw, 2019). Integrating architecture with the surrounding environment necessitates awareness and caution in order to respond to a common identity rather than individual preferences (Lambe and Dongre, 2019).

Amirshekari and Pourmand (2015) stated that new design and development in a specific space must be tied to its context. Contexts are typically established in a variety of scales that are suitable with the scope of architectural design. Topography, vegetation, urban conditions such as building density, street, sidewalks, and their relationships, types, and arrangements of materials, building distances, regional geography conditions, urban traffic density, and population are examples of contexts. According to Thomas and Garnham (2007) architecture must be located in a setting with historical and cultural contexts, and architecture must also combine the past and the present, as well as forecast the future. Marcantonio (2010) mentioned that new designs must be compatible with local architectural styles.

The peculiarities of the architecture, in terms of spatial characters, elements used and behavioral patterns, give a sense of place and identity to these areas, with the growing development and concern for the search for an identity, there is a need to revive the age-old qualities to fulfill the contemporary needs of society. Understanding the very essence of the built environment and responding to it sympathetically is the crux of the contextual design approach. Context theory, as described by Frampton (1983), the theoretical foundation for this work is provided by Lefaivre and Tzonis’s discussion on Contextualism in the twenty-first century (Lefaivre and Tzonis, 2020).

### 2.2 Procedural modeling

Procedural modeling refers to a group of generative techniques which can (semi) autonomously generate a certain type of content given a set of input parameters. It is used in a wide range of applications, including texture modeling, plant, building, urban areas, terrain modeling, road network, and art creation (Ghorbania and Shariatpour, 2021).

Lindenmayer created the Lindenmayer system (L-system), which was the first formal specification of procedural modeling, describing it as a parallel rewriting system used to construct strings, a collection of production rules used to generate geometric structures (Lindenmayer, 1968). Guo et al. (2020) proposed several procedural models based on L-systems to represent the algorithmic nature of many plant species. Meanwhile, procedural modeling became very popular in other fields of computer graphics, such as noise generation (Dylla et al., 2008), texture synthesis (Dylla et al., 2008; Ebert et al., 2002), and fractals (Luan et al., 2008). Within the last few years, procedural generation of architecture and buildings has become a popular topic in architectural auto modeling, due to its simple parameters and quick generating speed of a huge number of designs with comparable patterns and interfaces (Yong et al., 2012). In architecture, it originally appeared with shape grammar method by Stiny and Gips which was effectively utilized for analyzing the architectural design and new style of construction (Stiny and Gips, 1971).

Birch described a set of techniques that would enable historical building processes, from ancient Rome to modern Hong Kong, due to their role in rapid modeling and overcoming the sensation of repetition that plagues procedurally established environments (Birch et al., 2001). Additionally, Parish and Müller (2001), continued their extensive work in the architectural procedural modeling field, they proposed a procedural system for modeling city maps, geographical features, and buildings by using L systems.

Müller and Wonka kept working on procedural building creation methods, they introduced the Computer-generated Architecture (CGA) shape for the first time. CGA shape is an architectural procedural modeling grammar that produces high-quality, low-cost models. In the same year in another paper, they demonstrated how the CGA may be used to reconstruct architecture from the Puuc style found in the Mayan site of Xkipché, Mexico, by identifying Puuc building types and general designs and using them to generate sets of rules for its shape grammar (Müller et al., 2006). Other researchers have built on this method of grammar development through categorization and rules (Das and Garg, 2011; Dylla et al., 2008). Teoh (2009) developed a procedural modeling system that allows users to change parameters to create a variety of buildings in the style of ancient East Asian architecture, Teoh’s technique also allows artists to change the appearance of the structures using "custom-designed geometry", such as doors, brackets, beams, roof finials, and ridges, in addition to the specifications.

### 2.3 Augmented reality in architecture, construction, and engineering

Augmented reality (AR) is a technology that superimposes computer-generated information onto a user’s perspective of a real-world scenario. AR applications are growing increasingly varied and popular as AR technologies mature and become more well-established. The use of AR in practical applications such as education, design, manufacturing, construction, and entertainment shows enormous promise for improving existing technology and improving people’s lives (Delgado et al., 2020).

The first outdoor augmented reality application was developed by Feiner et al. (1997), dubbed the roaming machine, which combined augmented reality concepts with mobile computing mobility, with the goal of facilitating the interaction of ordinary people with the real world, which at the time entailed the use of a backpack containing a computer and several sensors. Afterward, this system was expanded to show 3D models of buildings as well as user-defined paths (Höllerer et al., 1999). Thomas et al. (1999) developed the Tinmith system to create augmented reality in-situ visualizations of building alterations, they employed a computer-aided design tool to model the original buildings and the new improvements, as well as GPS to register the content with the surrounding environment.

In the field of architecture and archeology, De la Fuente Prieto et al. (2017) presented a study in which they linked augmented reality and architecture fields through the concept of representation. Fonseca reported their experience in designing, developing, and evaluating new didactic methods, in order to help students to improve their spatial and graphical skills. Specifically, the use of Augmented Reality (AR) in architecture, urbanism, to build construction and interior design of undergraduates, and to master students learning processes (Fonseca et al., 2012). Virtual tours and in-situ walkthroughs have both benefited from AR. Kim et al. (2009) proposed an augmented reality system allowing tourists to immerse themselves in cultural heritage locations. Tan and Lim (2017) introduced an augmented reality virtual tour system to enhance the exploration of historical sites. Cao et al. (2019) conducted a comprehensive analysis of AR in the fields of Architecture, Engineering, and Construction, and Chu et al. (2018) investigated how AR might be utilized to improve information retrieval from BIM models, saving time in construction planning tasks. Mutis and Ambekar (2020) investigated the challenges of using augmented reality to execute project walkthroughs and provided a system that can visualize future construction site interventions. A detailed methodology of mapping AR into the specific activities in construction can be found in (Carreira et al., 2018; Chalhoub and Ayer, 2018; Chen and Fragomeni, 2019). Fazel and Izadi (2018) presented a novel affordable interactive multi-marker augmented reality tool that helps construction workers build intricate double-curved brick walls, two digital cameras, a head-mounted display, a CPU, and two markers that indicate the correct position and orientation of the bricks make up the proposed tool. Chalhoub and Ayer (2018) introduced an AR system that assists workers incorrectly installing electrical installations. Electrical conduits are modeled in 3D and superimposed in the exact location in the room, eliminating the need for 2D drawings.

Augmented reality was also used to support the design process, Kurmann (1995) developed computer software that allows for 3D virtual modeling in architecture, Sculptor, which focuses on the early stages of the design process, such as massing studies, as well as the creation of natural, intuitive, and direct virtual reality user interfaces. Sandor and Klinker (2005) introduced an AR system called ARCHIE, that allows architects to collaborate on a virtual model placed on a table and show the design intent to stakeholders. Sareika and Schmalstieg (2007) developed an app to promote and improve urban design discussion, a live video feed shows the location of a tent where people are congregating to discuss potential improvements to the region. The interaction takes place on a giant projection screen that is visible to everyone, and users can utilize augmented reality to create real-time sketches over the video broadcast. Langlotz et al. (2012) suggested solutions for both local and large environments, by presented a novel approach for mobile Augmented Reality content development in unprepared contexts. Nee et al. (2012) highlighted how augmented reality may be used to enhance collaborative design by allowing multiple users to engage with a single virtual model rather than requiring physical mock-ups. Despite all modern technologies that provide photorealistic models on stereoscopic monitors and screens, there is still a perceptible distinction between virtual and real-world structures. Song et al. (2021) presented a study to improve the immersion level of urban planning solutions, and how to use augmented reality to allow urban planning experts moving around city streets and project virtual three-dimensional buildings, allowing simultaneous viewing of the real city and virtual buildings.

## 3 Methodology (our approach)

**Figure 1:**
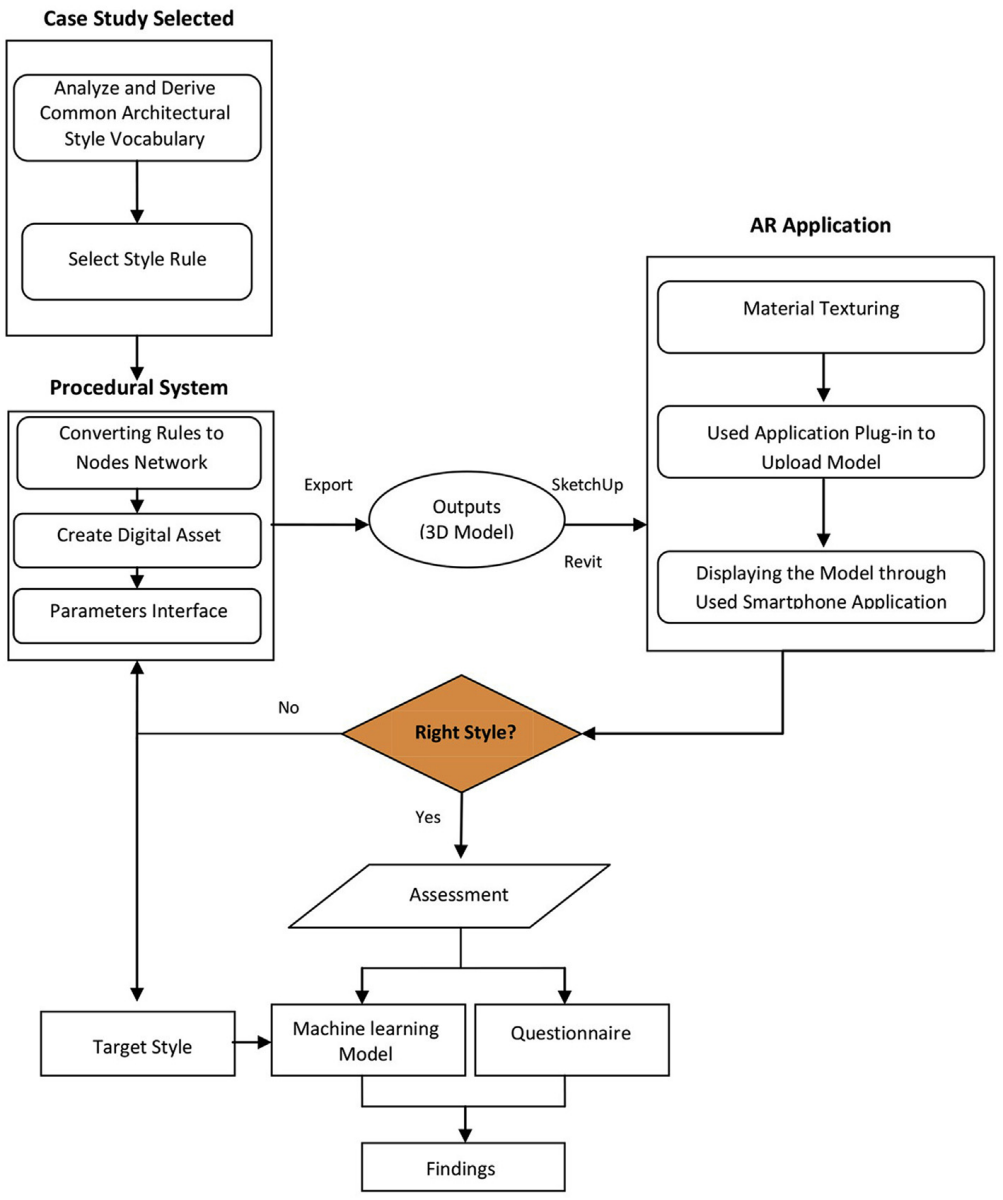
The proposed approach workflow.

Here, we discuss a new contextual design approach that combines the power of procedural modeling with the ease and speed of modification through the control of certain parameters and the use of augmented reality to address the challenges of residential building identity in Amman and the influence of Western movements. To improve the design of complex structures, we analyzed study samples and derived the most important common architectural vocabulary, then transformed them into a network of nodes and parameters through which buildings belonging to the previously analyzed study samples can be generated, followed by imposing the generated buildings on the real world using an augmented reality application to facilitate decision making and to check the building’s integration with the surrounding context. Our approach is broken down into numerous steps, as illustrated in Figure 1.

### 3.1 Case study (The residential buildings in Amman city)

**Figure 2:**
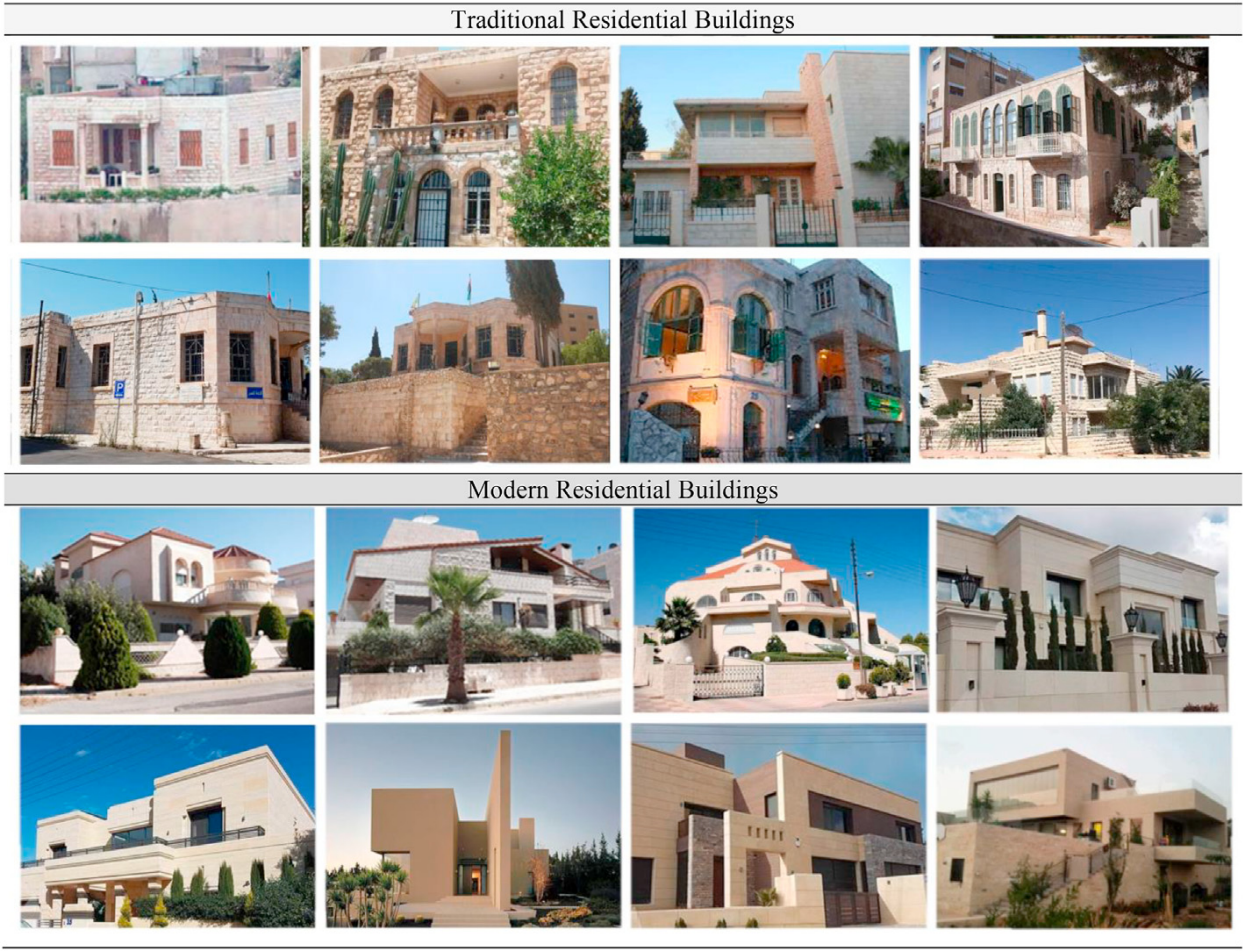
The stylistic variety of Amman. (Dahabreh, 2020; Assarai Engineering Firm, 2022; "Abu Samra House/Symbiosis Designs LTD" 13 Apr, 2011).

Amman was selected as a case study in order to address the issue of Western architectural movements influencing the architectural style in Amman, which is incompatible with the city’s identity and its context (see Figure 2).

#### 3.1.1 Main features of Amman residential buildings

Amman has three main characteristics that distinguish it from other cities which have greatly influenced its development over time: firstly, while Amman is a relatively recent city, the land on which it was built has a lengthy history. Secondly, the diversity of the city’s population, which has been and still to be formed by numerous waves of displacement and asylum movements as a result of regional wars, as illustrated by Palestinian refugees who arrived in 1948 and 1967. Followed by the Gulf War of 1991 and the return of most Jordanian employees from Kuwait and Iraq. The Iraqi migration in 2003, and the migration of Syrian refugees in 2011, this has formed the city’s identity more than any other dynamic feature. Finally, the Lack of social and urban cohesion as a result of failed planning attempts due to the city’s rapid expansion. These three characteristics played a crucial role in forming the architectural identity in the city of Amman (Dahabreh, 2020).

Different types of residential buildings evolved in the city of Amman under different geographical, climatic, and traditional features. Despite of this, all the residential buildings have certain characteristics and features in common (Daher, 2014). In Amman, most residential houses consist of one level, the floor space of the houses began to expand over time (Al-Azhari, 2020), as it began to expand vertically and the number of floors increased. The most important characteristic of residential buildings is the plan layouts of the houses. In general, buildings in Amman can be classified into two sections (Shawash, 2003), traditional residential buildings: the three-bay house type (the Iwan-like model, and the Gallery model), and modern residential buildings.

The residential buildings of a three-bay model were influenced by the modernity that leaked to Amman at the late Emirate period. Despite the great change that it underwent, it remained in the order of three-bay arrangement. For example, there is no longer division between floors into ground floor and ‘piano nobile’, as each floor now forms an independent household in its own right, and individual houses usually consist of one floor only (Shawash, 2003), these buildings were built for wealthy notables, using imported materials, and local labor. The good examples of these houses that represent the evolution of modernism in the city of Amman are the White Palace, Tabba' house, and Balbeisi house. They also introduced phenomena that attained great recognition in Amman’s architecture, such as the semi-circular room, which is evident in the Dubbati house (Shawash, 2003).

From the 1960s through the mid-1970s, a domestic contemporary architecture style emerged, leading to what has been dubbed "domesticated modernity", which is the amalgamation of modernist influence with the local constructing traditions of masonry and material (Daher, 2008).

From the 1970s to the mid-1980s, two architectural trends predominated in Amman city: one is an eclectic cornucopia of designs which have demonstrated luxury and financial power, while the other is a kind of picturesque Islamic revival that expresses nostalgia the past, these trends represented a condition of confusion in a culture that is both dogmatic and indefinable, in the sense that it is neither traditional nor contemporary in the modern sense, but rather a malformed hybrid (Dahabreh, 2020).

From the mid-1980s, alien architectural phenotypes imposed their impact on architecture and threatened the morphology of Amman city, and its urban tissues, with the emergence of monumental facades, glass and steel buildings (Goussous and Qashmar, 2019). More recently, Amman has seen more diversity in its architectural style, ignoring its true cultural and regional identity, this may be due to rapid urbanization, technological advances and the rapid spread of digital media. All this contributed to the detachment of its inherited morphology from the most recent architectural developments (Rababe’h, 2010).

#### 3.1.2 Selected samples analysis

**Figure 3:**
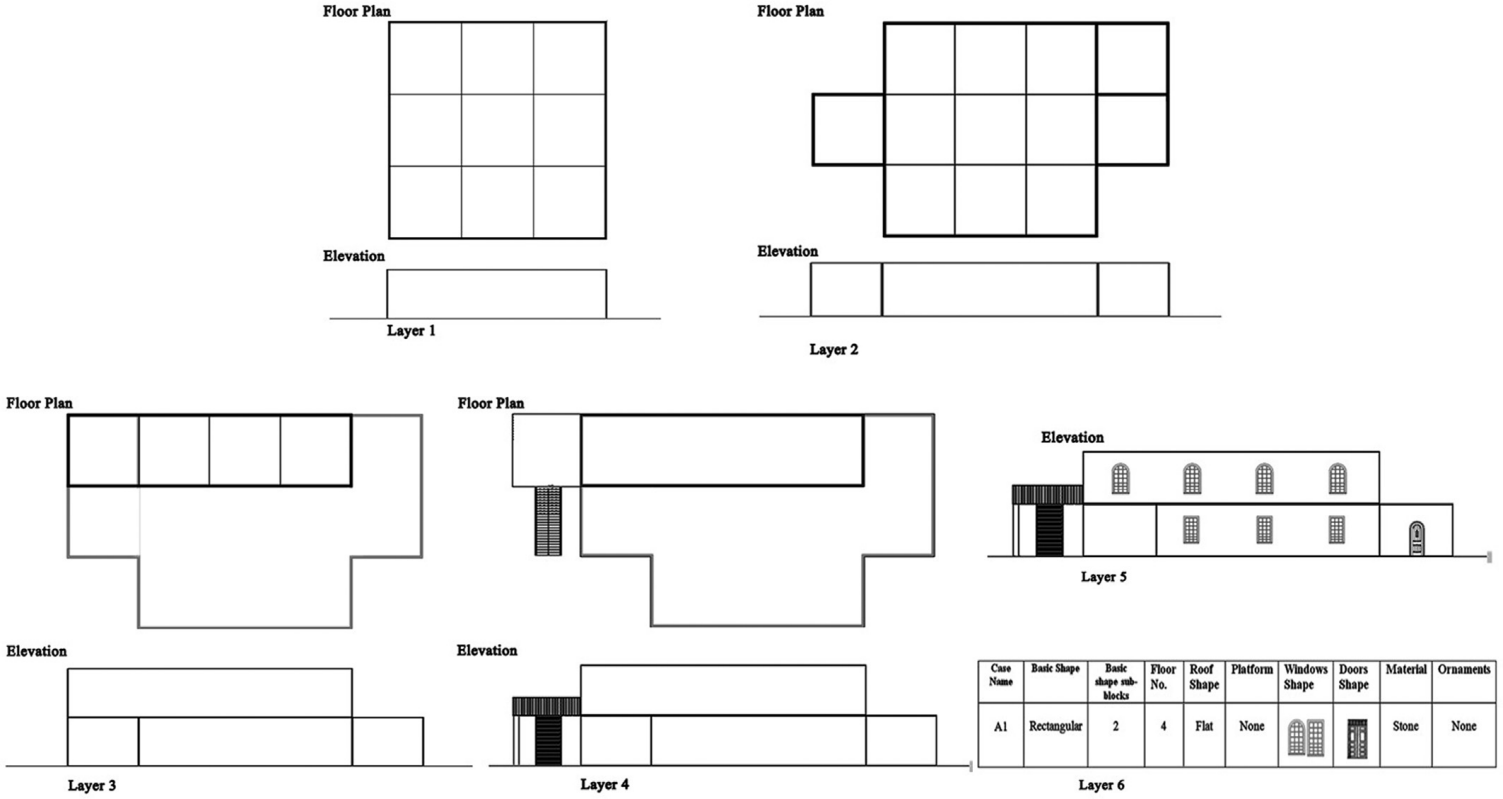
Layers of abstraction/cumulative complexity.

To create a procedural system for generating buildings in Amman, we must first create an appropriate model for describing and classifying the primary characteristics and vocabulary of Amman’s residential forms. The analysis began with each residential building’s exact details being recorded, and then each building was subjected to numerous levels (degrees) of abstraction to register its attributes at each stage of construction. The following method was used to conduct a pilot analysis at various levels of generality for the assessment of architectural buildings, so that they were examined at the level of complexity and the information gained was gradually reduced in a backward process until their very basic initial form was revealed, and how the initial form evolved to produce the final facade. Finally, the following stages of abstraction/cumulative complexity were used to build this analyzing method, as shown in Figure 3.

Layer 1: The architectural composition of the building is outlined in this first layer to clarify the basic shape which generated the form.
Layer 2: This stage enhances the articulations on the basic masses of the building, so that the major volumetric alterations within or on the basic masses and resulting in an addition to or subtraction from the basic form are presented.
Layer 3: This stage enhances the vertical expansion on the building’s fundamental masses.
Layer 4: This stage reinforces the perception of variety across buildings; by adding some structural and architectural elements such as platforms, stairs and railings.
Layer 5: This stage focuses on the appearance in greater detail, including the addition of basic piercing(s) of the facade’s structure, as well as the selection and organization of geometric descriptions of piercing(s), and the contextual relationship of piercings with each other and with the surrounding.
Layer 6: Along with these five stages, a sixth layer has been created to further characterize and categorize the preceding qualities. Several successive sub-categories were created within this category (window, door shapes, etc.).

In this section, 6 samples were selected and their characteristics were discussed in Table 1, in terms of building shape, details (windows and doors), and exterior (stairs and platforms).

**Table 1:**
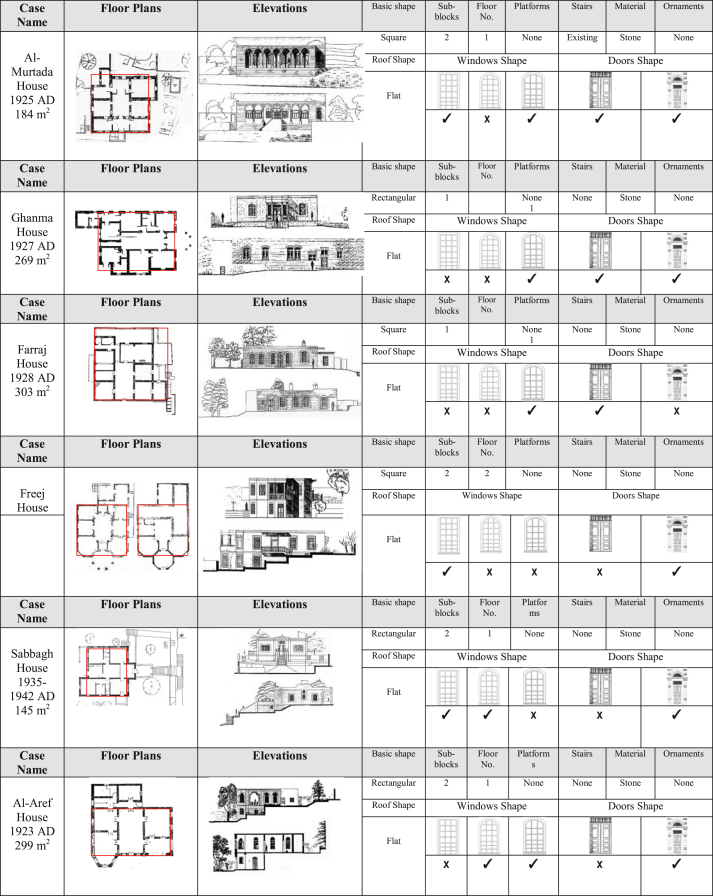
Analysis of the basic elements of the selected samples. Figure source: Rifa’i, 1987.

Table 1 demonstrates that there are a number of constants for all study samples such as the shape of the basic unit, the flat roof, and the building material is limited to stone and no ornaments are used. The buildings' layout, the number of doors and windows, and their shapes, as well as the use of platforms and stairs, are all variables.

### 3.2 Creating procedural workflow

In this study, Houdini software was used to create a procedural system by generating node networks that can be turned into a reusable custom node, a Houdini digital asset (HDA), with its user interface for parameter control (SideFX, Kramer and Akleman, 2020). Our HDA’s interface was created so that the users can easily understand what they’re doing of the model. The portions of the parameters are divided into shape, details (windows and doors), and exterior (stairs and platforms). The interface is additionally organized by hiding certain parameters until a qualified parameter is toggled on; for example, the platform placement parameters are only shown when the user switches stairs, this lowers the interface’s visual clutter.

The procedural generating parameters allow for complete customization of the buildings, as well as taking into consideration some actual elements, such as the fact that the door and the window cannot be placed in the same location. These characteristics also allow the user to produce a wide variety of options, allowing him to select the desired combination of features for the building rather than being forced to select combinations that are likely to be found in realistic Amman buildings.

**Figure 4:**
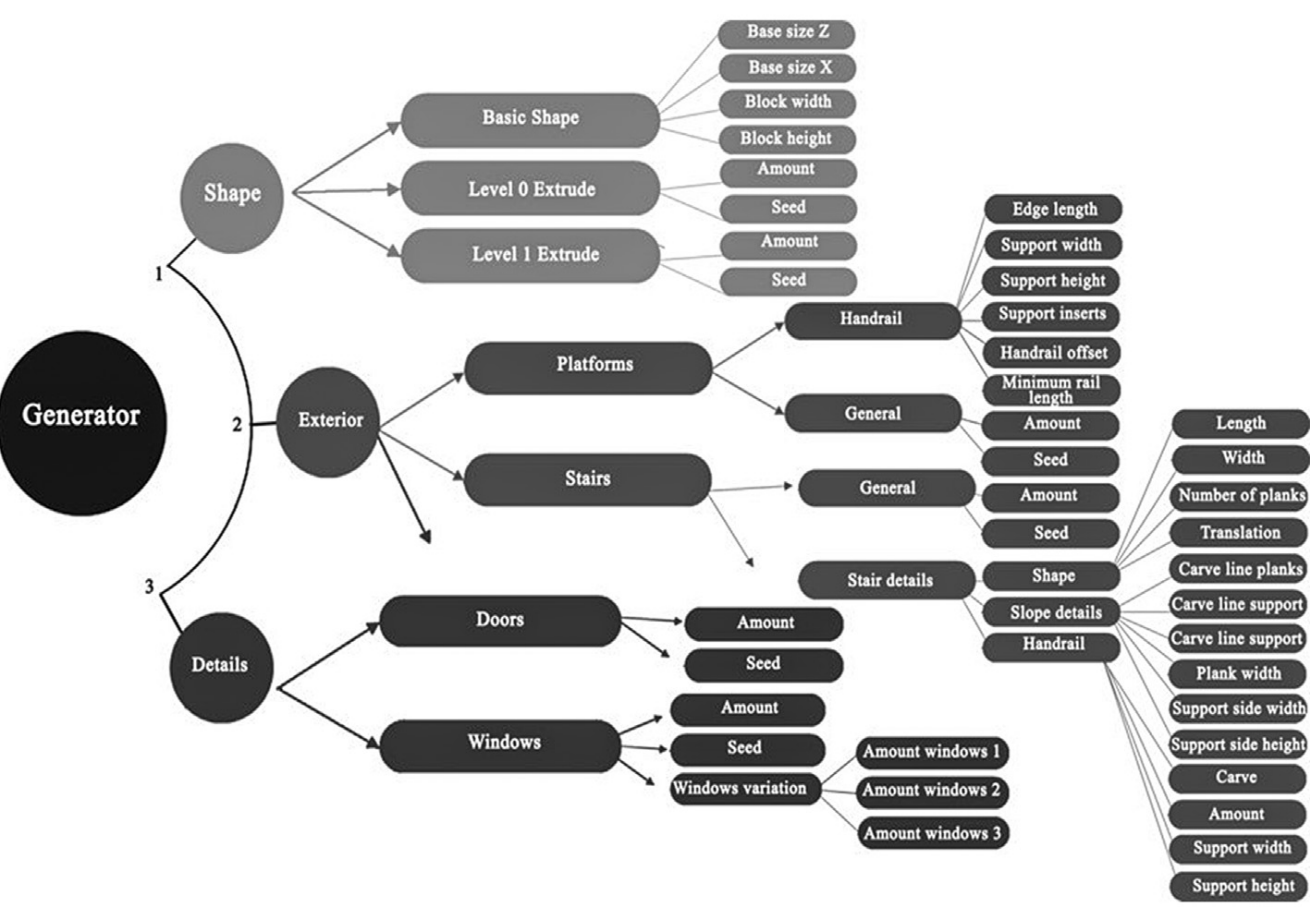
Procedural system workflow.

The generated digital asset generates buildings by iterating on layer iterations, resulting in a trees structure of building components. As seen in Figure 4, the procedural system workflow is summarized.

#### 3.2.1 Shape

**Figure 5:**
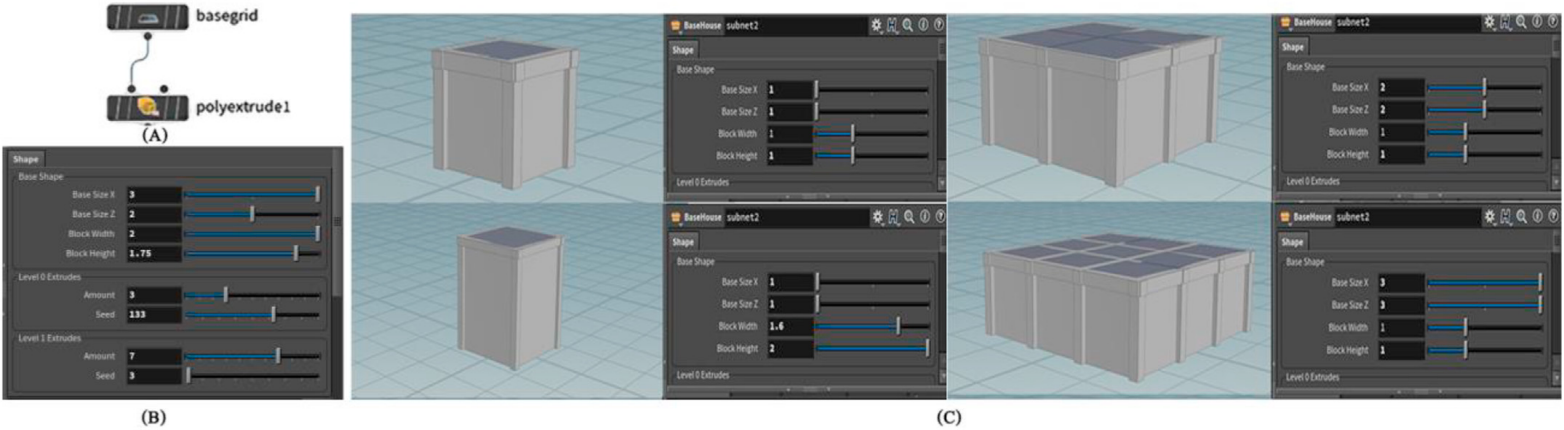
(A) Nodes network to generate basic shape asset, (B) The interface of basic shape parameters, and (C) shows some configurations can be formed by controlling basic shape parameters.

The main portion of the building is the shape, on which the other layers are created. Since the majority of residential buildings in Amman have a regular shape, the basic block was chosen to be square in shape and can be repeated on the Z and X axes, to obtain the required basic shape. The user can define several characteristics to alter the appearance of the building’s fundamental shape, such as length, width, and height. These parameters are block width, block height, base size x, and base size z, as indicated in Figure 5.

**Figure 6:**
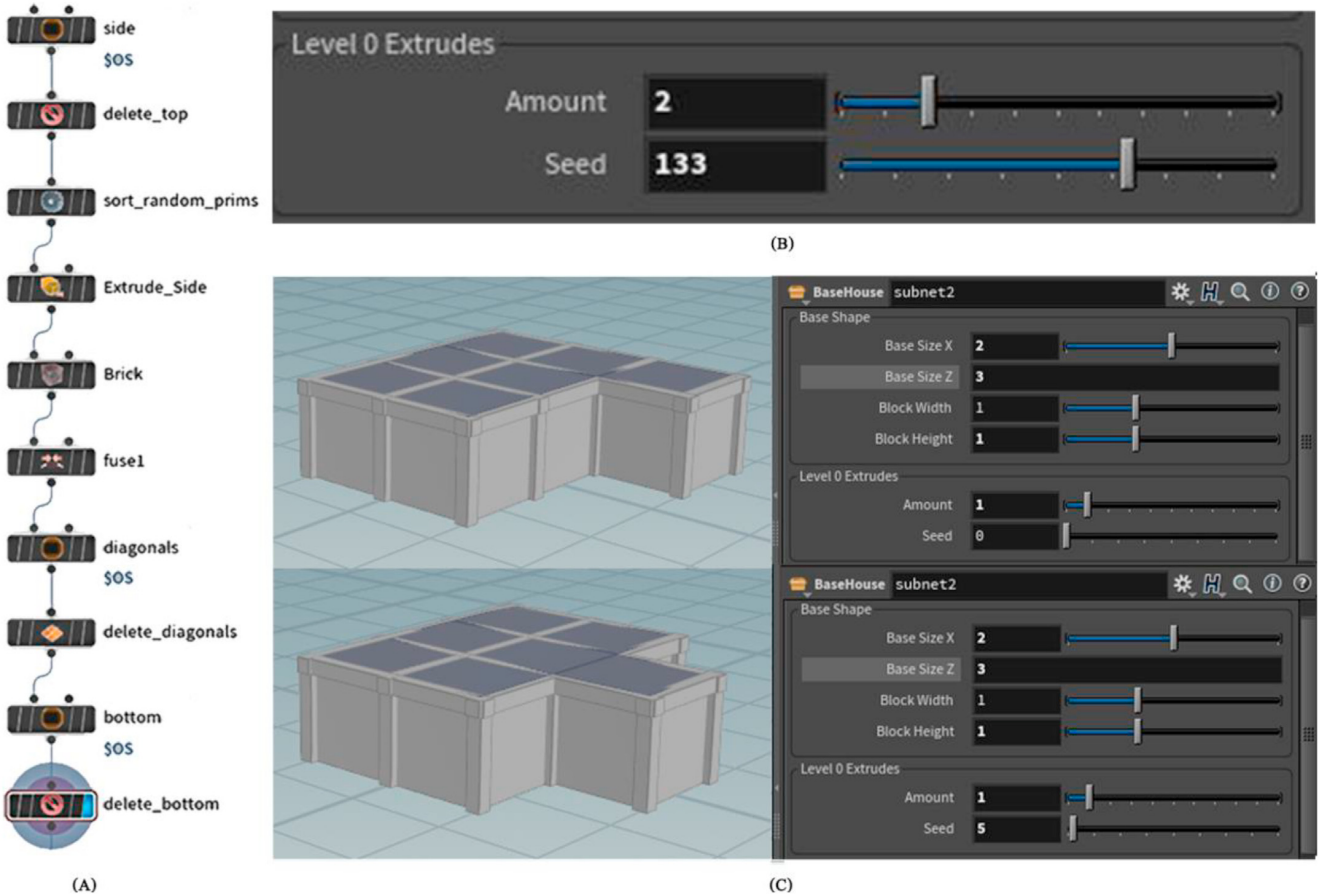
(A) Nodes network to generate level 0 extrudes asset, (B) the interface of level 0 extrudes parameters, and (C) some configurations can be formed by controlling level 0 extrudes parameters.

**Figure 7:**
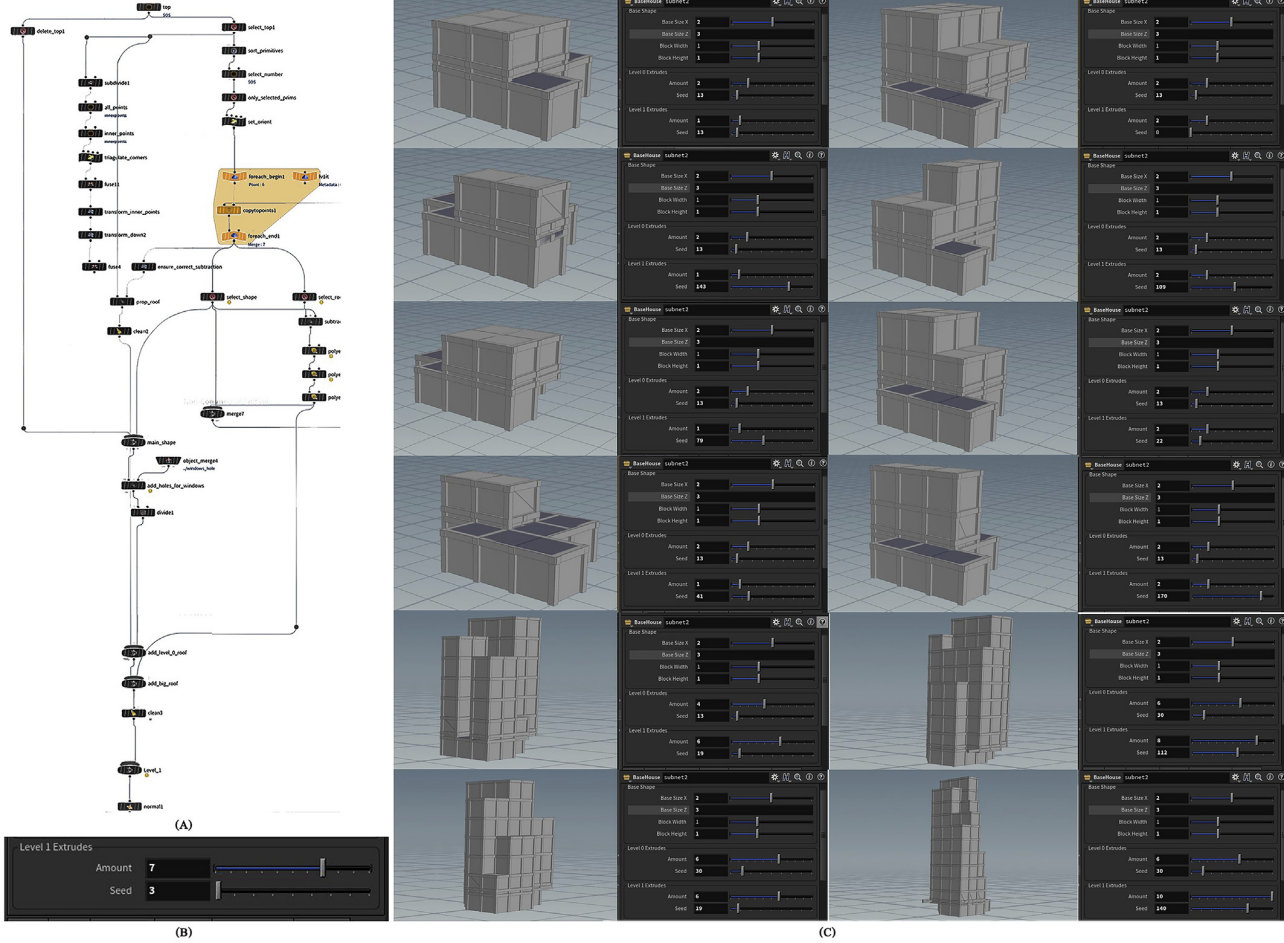
(A) Nodes network to generate level 1 extrudes asset, (B) the interface of level 1 extrudes parameters, and (C) some configurations can be formed by controlling level 1 extrudes parameters.

Sub-blocks emanating from the basic shape can also be added using the Level 0 extrude parameters, which can be used to determine their number and location on the basic shape, as seen in Figure 6. The user can create levels and set their number, and composition using the Level 1 extrude parameters, as shown in Figure 7.

#### 3.2.2 Exterior

**Figure 8:**
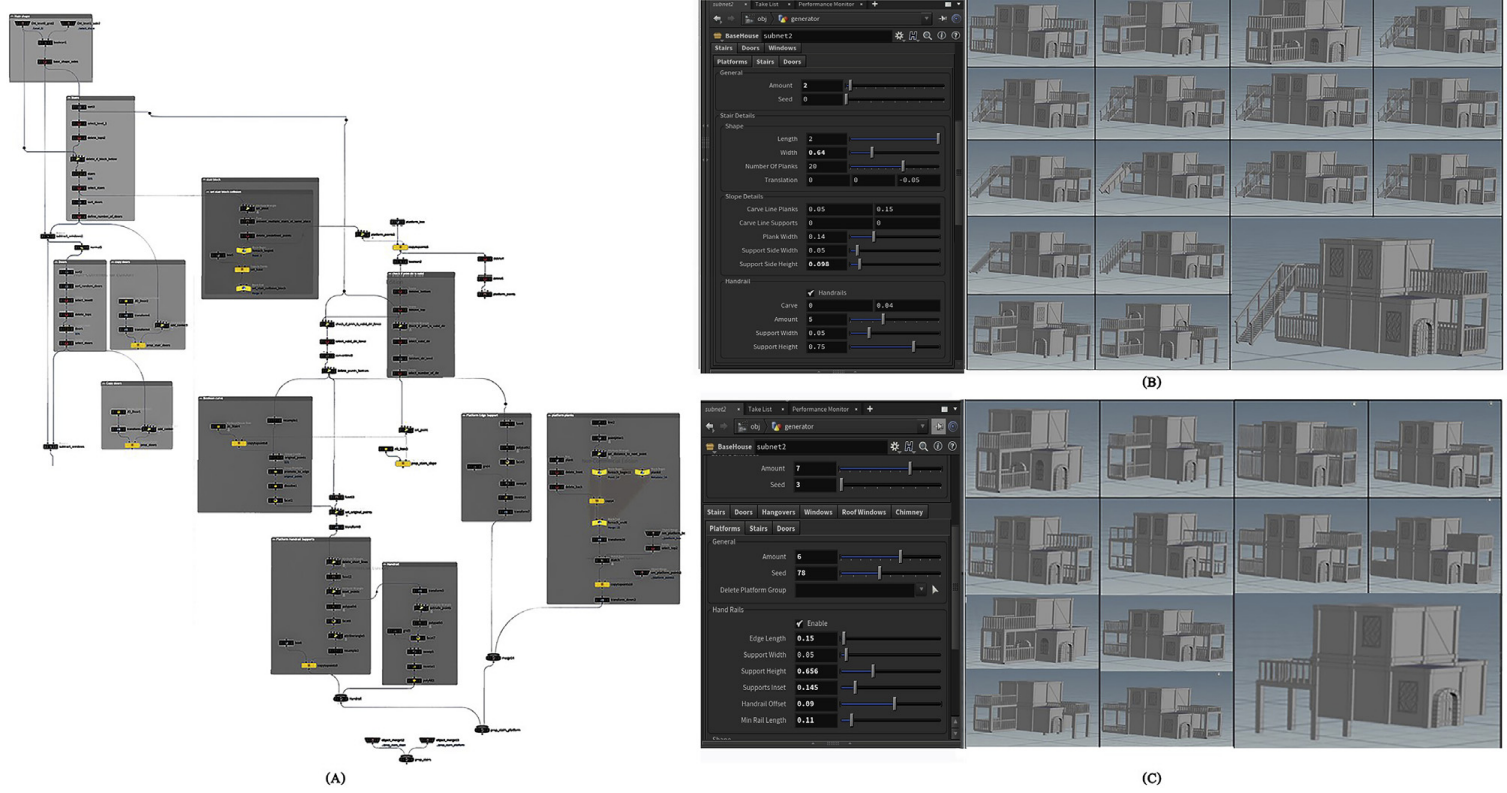
(A) Nodes network to generate platforms and stairs asset, (B) the interface of stairs parameters, and some generated configurations, and (C) the interface of platforms parameters, and some generated configurations.

Some structural features, such as platforms and stairs, can be added to the exterior layer. Platforms are architectural elements that can be added to a building’s front, left, and right sides. Some of its general aspects, such as the number of platforms and their distribution, can be defined by the platform’s parameters. In addition, the edge length, support width, support height, support inserts, handrail offset, minimum rail length, and design of the platform handrails must be specified, as illustrated in Figure (8).

Aside from the option to add some elements, such as whether the platform is attached to a stair or a door, and the possibility of specifying the characteristics of each of them separately. Stairs often lead to a side entrance to the house, by the stairs parameters, the number of stairs and their distribution can be set using the stair’s parameters In terms of shape, manipulating the variables of length and width, number of planks and translation, and in terms of slope details, determining the value of each of the following variables: carve line planks, carve line support, plank width, support side width, and support side height are all useful. In addition, the properties of the handrails can be added and modified using the following parameters: carving, quantity, support width, and support height, as illustrated in Figure 8.

#### 3.2.3 Details

**Figure 9:**
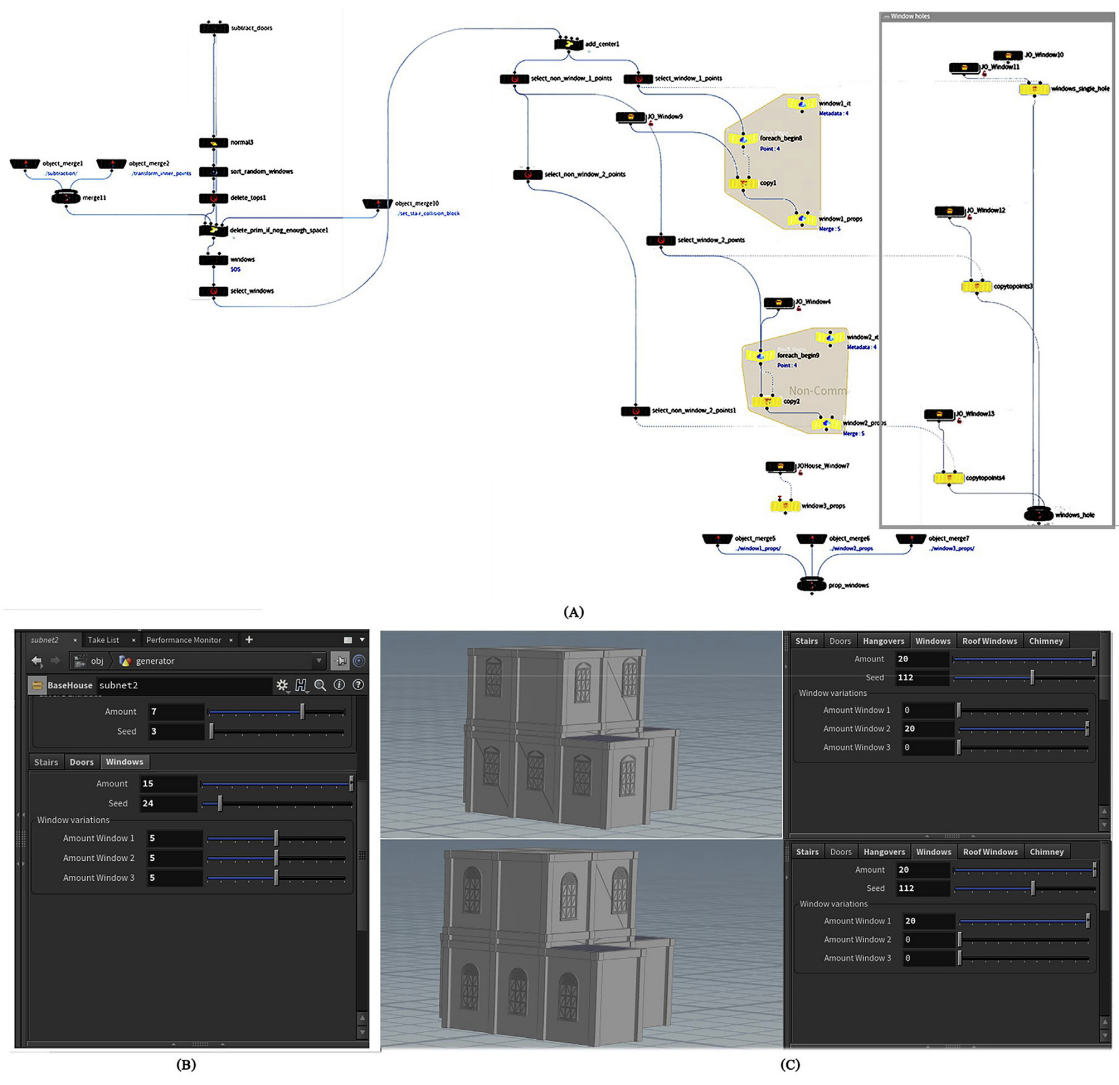
(A) Nodes network to generate windows asset, (B) The interface of windows parameters, (C), Some configurations can be formed by controlling windows parameters.

The basic opening(s) of the facade’s structure, whether they were doors or windows, as well as the selection and organization of geometric descriptions of opening(s), and the contextual relationship of openings with one other and with the surrounding, are all included in the detail layer. By controlling the parameters of windows asset, the user can specify the total number of windows and their distribution locations, as well as the type of windows to be used, such as an arc window, window with an ellipse top or a rectangular window, which is the most common window shape in Amman, with the option of merging more than one type and determining the amount of each, as shown in Figure 9.

**Figure 10:**
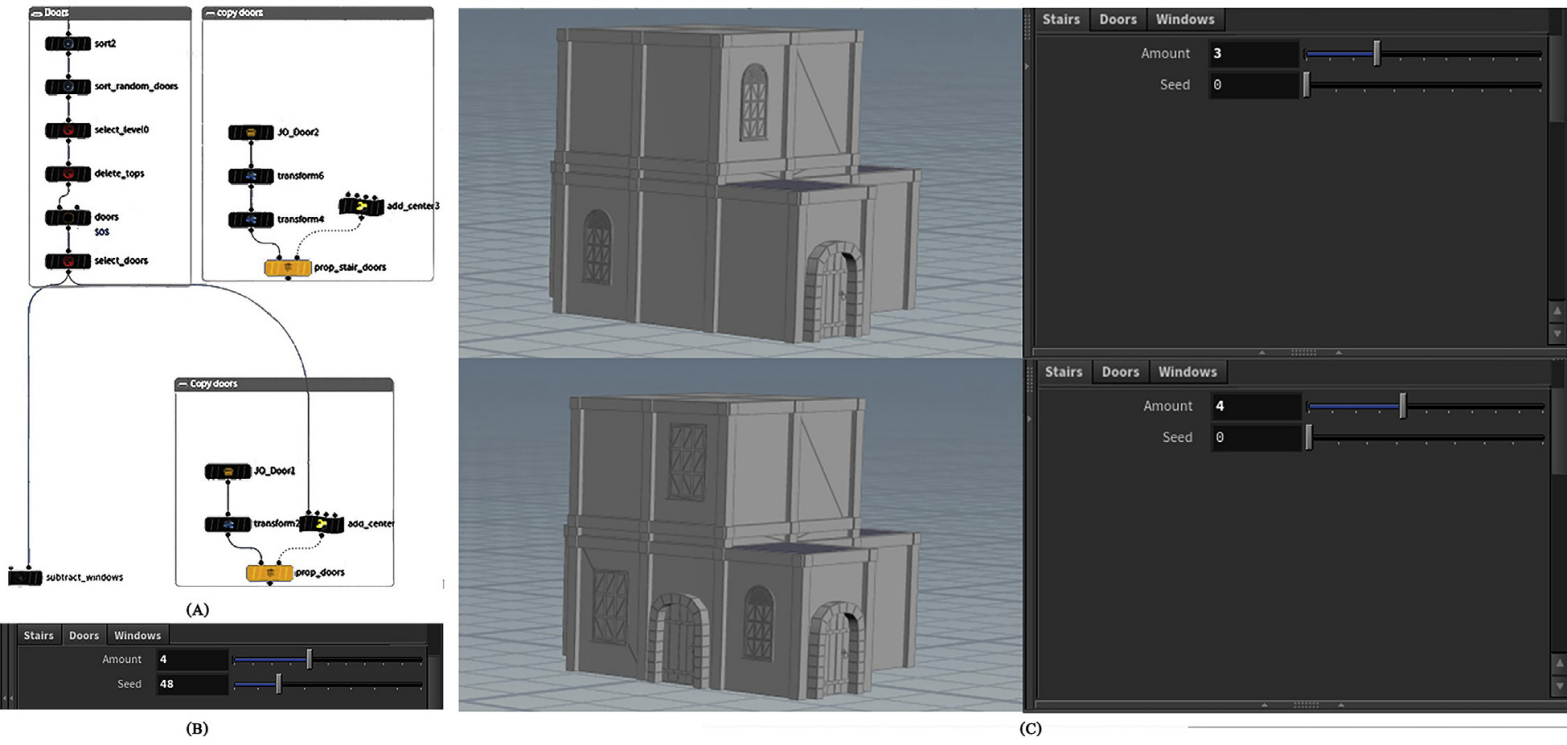
(A) Nodes network to generate doors asset, (B) the interface of doors parameters, (C) some configurations can be formed by controlling doors parameters.

Next is another feature that is always present, the door. The number of doors and their position can be determined using the door asset’s parameters, ensuring that the door and window do not overlap, as shown in Figure 10.

### 3.3 Augmented reality (AR) application

Our approach aids in the visualization of architectural designs in their physical surroundings. The architectural designs were displayed in relation to their physical condition on the site using the mobile application (Augin), which allows importing the design developed from architectural presentation applications after covering it, such as (Revit Autodesk, 2020 or SketchUp), and then presenting it.

**Figure 11:**
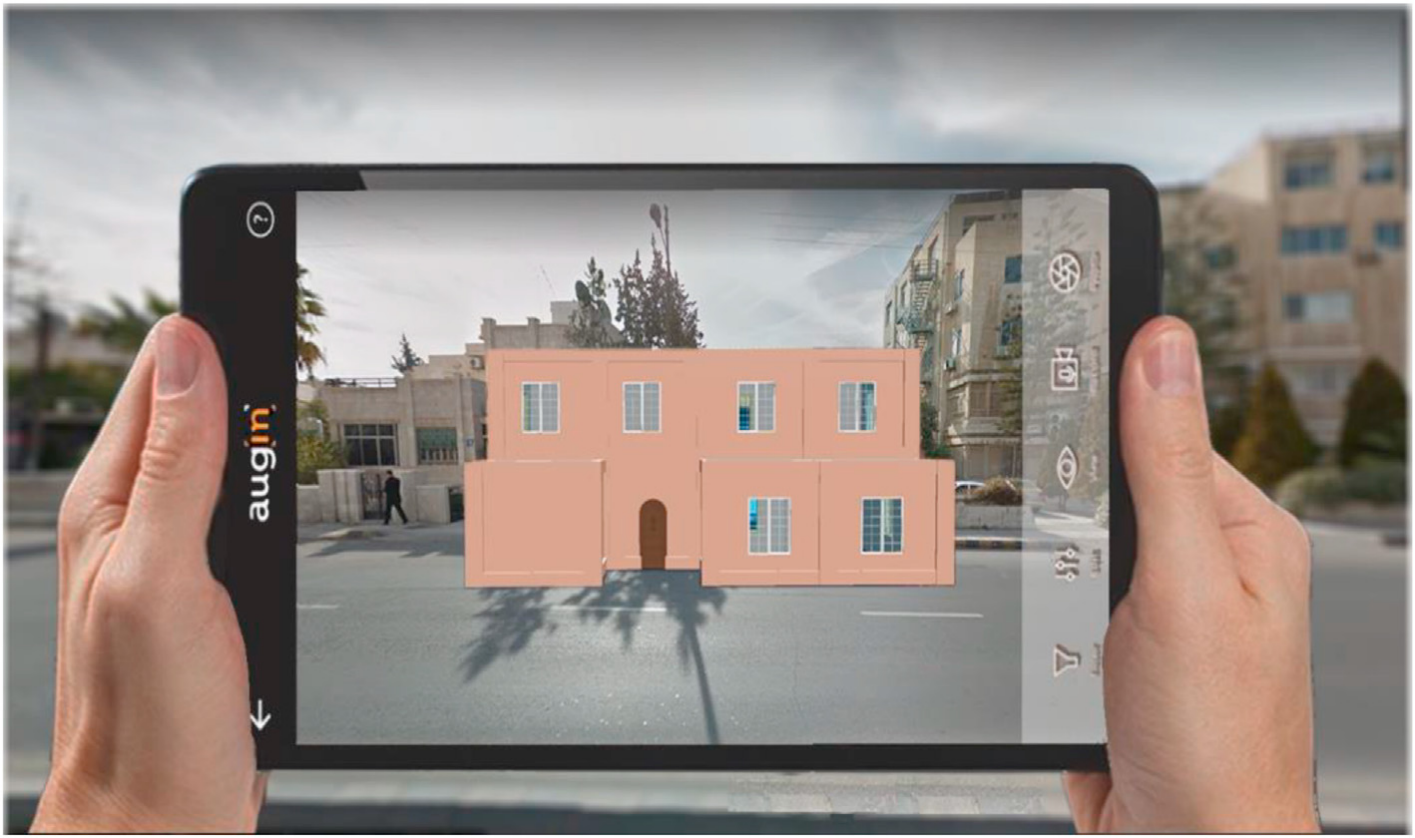
Display 3D model in real-time using the Augin application.

The ability to visualize or perceive components of an architectural design in the field is at the heart of the technology, providing the user a feeling of how the design fills or modifies real space. As shown in Figure 11. The use of procedural modeling in conjunction with the Augin application provides great flexibility, in that large, fast adjustments can be made in less than a minute using the parameters of the procedural system.

## 4 Evaluation and results

In our approach, creating buildings that are compatible with the surrounding context is a critical issue, so we considered using procedural methods combined with augmented reality in our task. We first used the procedural model system, which could generate semi-style and semi-structural for residential architecture in the city of Amman, and then adjust the details by a set of parameters. We also applied artificial intelligence techniques, specifically a machine learning model, to assess the compatibility of the generated designs with the architectural style of residential buildings in the city of Amman, thus producing designs that are in harmony with the surrounding physical context.

### 4.1 User study evaluation

In this section, we describe a user study to evaluate the effectiveness of our proposed approach, 30 architectural students were invited from the University of Jordan’s 4th and 5th grades, all of whom were familiar with computers but had no prior experience with procedural modeling or augmented reality technology. We spent 45 min training each participant in our proposed procedural modeling approach and another 30 min training them in augmented reality technology using free online tools. After that, the participant was shown a random sequence of photographs of residential buildings in Amman and old urban blocks.

Participants were required to use the proposed approach to design a residential building in the same architectural style as those depicted in the photographs. The participants began by using the researcher’s procedural modeling system, which was created using Houdini software, and then enhanced their designs by using the augmented reality technology, as well as making any necessary adjustments, for a total of 30 min per task.

The qualitative findings of the research experiment outlined in the preceding section will be analyzed and discussed. A research sample of 30 architectural students actively participated in testing the proposed approach, which integrates a procedural generator with augmented reality. The major purpose of this section is analyzing the tested components in order to attain the main goal of this research, which is to determine whether this novel approach is effective to create contextual design and spatial congruence.

**Figure 12:**
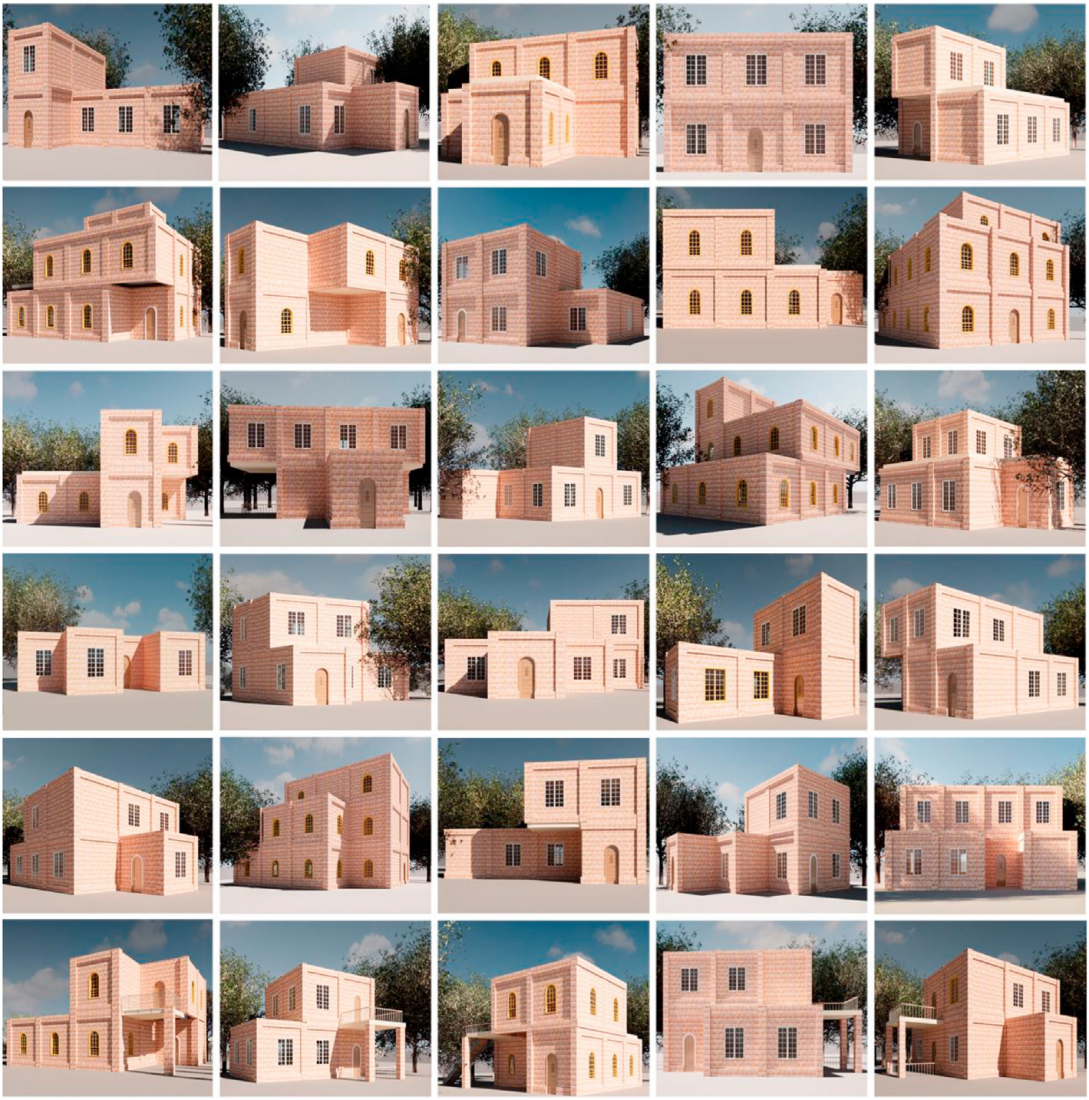
Students' designs that are based on the architectural style of Amman’s residential buildings.

The students were ecstatic to be a part of the experience, given the new technology procedural modeling poses to them, as well as their desire to experience augmented reality technology. The proposed approach’s outcomes, which were produced by the students, are structured in Figure 12. In this study, Teachable Machine, a web-based tool that makes creating machine learning models fast, easy, and accessible to everyone, was used to classification the proposed approach’s outputs (Teachable Machine).

**Figure 13:**
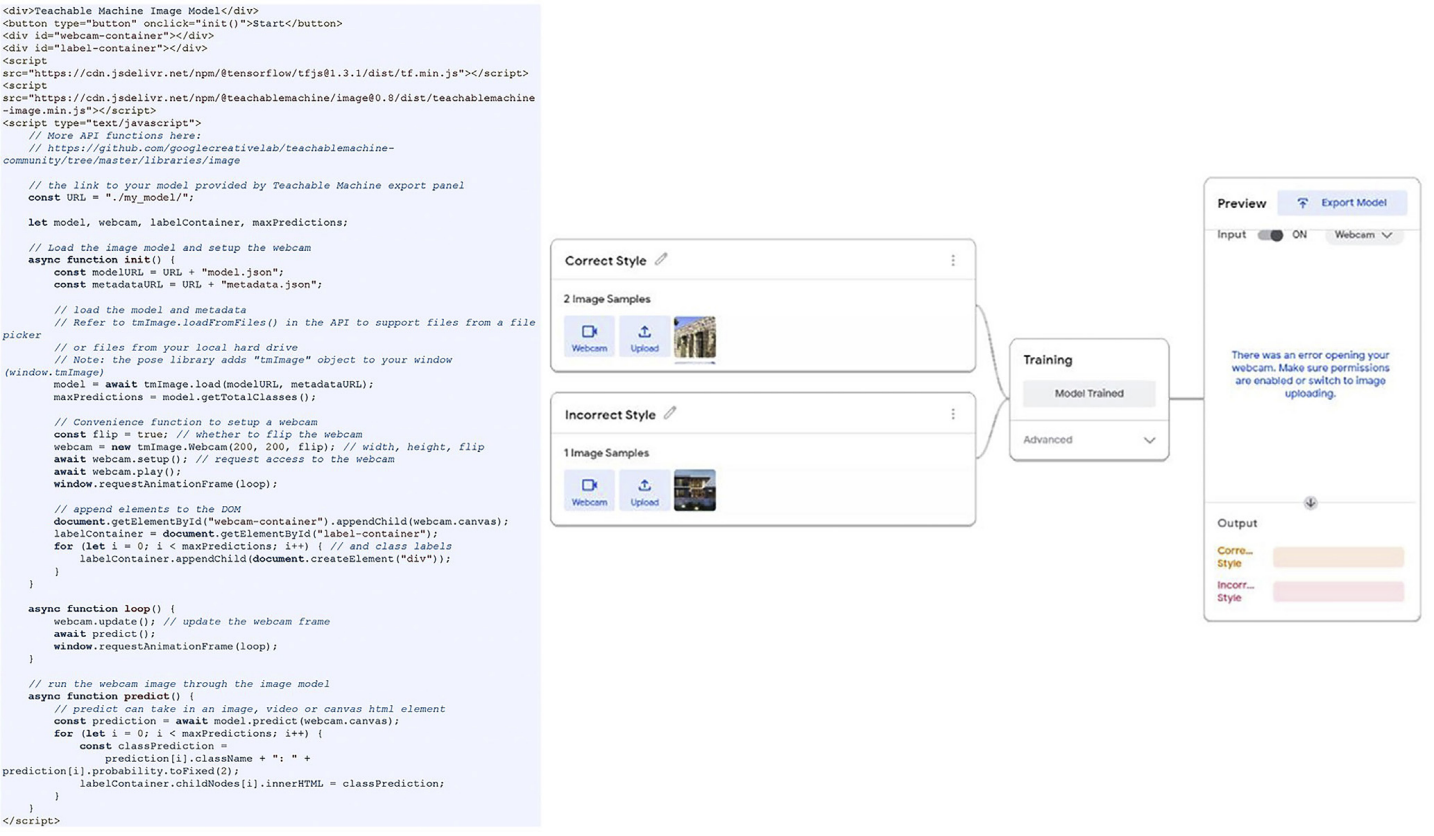
The machine learning model used.

**Figure 14:**
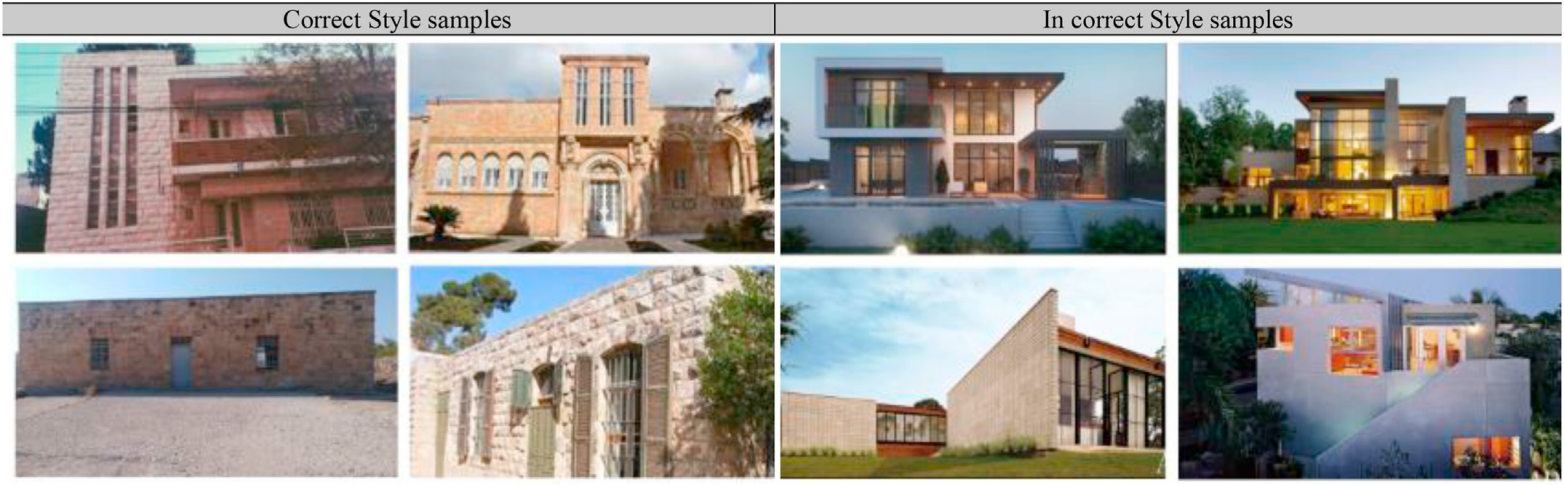
Some samples used in the machine learning model. (McGrath, 2017; archello, 2018; Tashatgo).

The machine learning model was created to assess how well the output designs of this approach match the context in which photographs are used. The model was first provided a set of photographs depicting the architectural style of residential buildings in Amman (correct style) and others depicting various modern architectural styles (incorrect style), and then it was trained to be able to classify the extent to which the proposed approach’s outputs are compatible with the architectural style of Amman, as shown in Figure 13 and Figure 14.

## 4.2 Discussion

**Figure 15:**
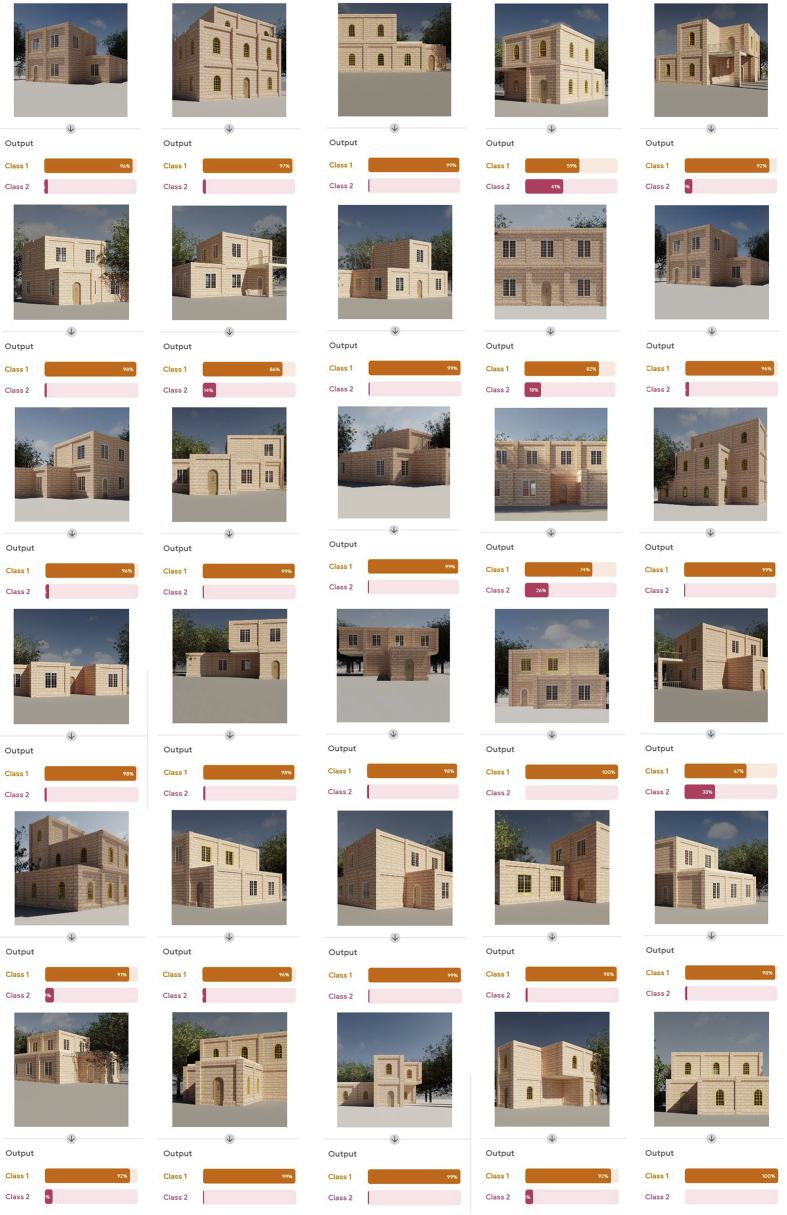
The proposed approach outcomes assessment using machine learning model, class 1 refers to correct style and class 2 refers to incorrect style.

The outputs of the proposed approach were checked and categorized using a machine learning model, as shown in Figure 15, and the findings were summarized in Table 2.

**Table 2:** Machine learning model classification findings. source: (Microsoft excel updated by authors, 2021).

Examination of the proportion of similarity of the produced designs to the architectural style of residential structures in Amman.										
Score	0–10	11–20	21–30	31–40	41–50	51–60	61–70	71–80	81–90	91–100
Designs No.	0	0	0	0	0	1	1	2	2	24
Avg.	92.5%									

The result showed that 24 of the outputs designs match the architectural style of residential buildings in the city of Amman by a percentage ranging between 90 and 100. While 2 of the designs achieved a percentage ranging between 81 and 90, and 4 designs achieved a percentage ranging between 50 and 80. 59% was the lowest percentage obtained. As a result, with an average of 92.5 percent, the proposed approach can provide an integrated design with the surrounding physical context.

The findings obtained from the questionnaire which describes the attitudes and preferences for implementing the proposed approach filled out by 30 students are listed in Table 3.

**Table 3:** Structure questionnaire findings. The 30 responses are represented by rows, and the four aspects are represented by columns. The range of values is Strongly Disagree = 1 to Strongly Agree = 5. The percentage from the average of the answers out of 5 was used to compute the score. Source: (Microsoft excel updated by authors, 2021).

Questions that determine preferences and attitudes toward the use of Proposed Approach?																									
Question	Do you think that using procedural modeling enhances the ability to design creative models compared to traditional methods?					Do you think the procedural modeling method facilitates the process of modeling and revisions?					Do you think the procedural modeling method is an ideal solution to generate a large number of designs of a certain style?					Do you think viewing your building design in the exact location where it will be built helps you in making the decision in the design process?					Do you think that ​the integration of AR has a positive impact on the design process?				
Score	1	2	3	4	5	1	2	3	4	5	1	2	3	4	5	1	2	3	4	5	1	2	3	4	5
Students No.	0	0	0	10	20	0	0	3	9	18	0	0	3	12	15	0	0	2	16	12	0	0	4	17	9
Mean Ang.	4.696					4.478					4.348					4.348					4.391				
Mode	5					5					5					5					4				
Std. deviation	0.470					0.665					0.647					0.619					0.656				

**Figure 16:**
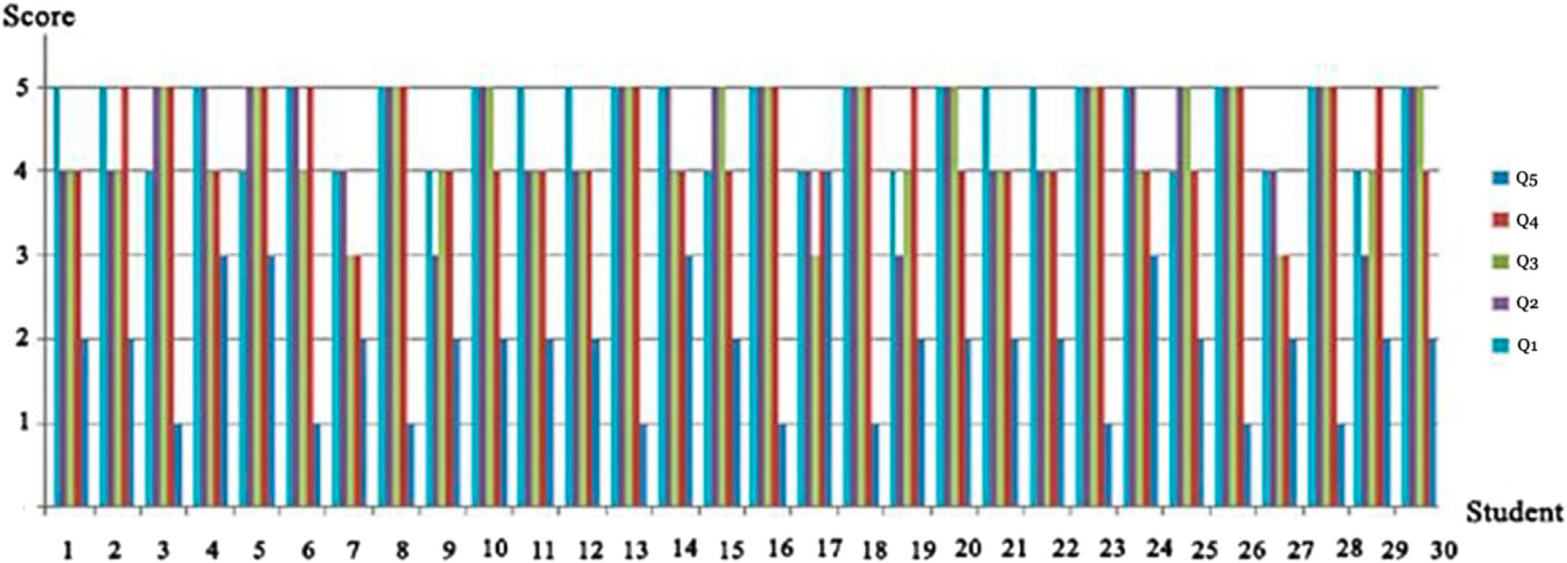
Students' responses to the questions which describe the preferences and attitudes toward the use of proposed approach.

As for the findings in Table 3, about the preferences and attitudes toward the use of the proposed approach the results showed that all the students agreed that procedural modeling improves the ability to design creative models when compared to traditional methods, with the highest score was 4.696 out of 5 on average. In addition, 27 of the students were between strongly agreed or agreed that it facilitates the modeling and revision process, and is an ideal solution for producing a large number of designs in a specific style, also most of the students believed that viewing the building in the exact location where it will be built helps them in making the decision in the design process, with the highest score was 4.348 out of 5 on average. As we are asked the students if they thought that the integration of AR has a positive impact on the design process; 26 of the responses were divided between agreed and strongly agreed, while 4 students were neutral, with the highest score was 4.391 out of 5 on average, as illustrated in Figure 16.

## 6 Conclusion and future works

The contextual design approach necessitates a deeper grasp of the physical surrounding and an analysis of the existing architectural patterns to derive rules and features that would assist create a harmonious environment. This paper presented a general framework for creating contextually suitable designs by integrating procedural modeling and augmented reality, in an attempt to start the process of obtaining spatial congruence. The architectural style of residential buildings was investigated using shape grammar-based procedural modeling. Procedural modeling is a methodological approach that functions as an analytical tool for studying existing architectural styles and developing new designs.

Given the impact of western architectural movements on Amman’s architecture, which has resulted in contemporary constructions that are aesthetically unsuitable and unsympathetic to existing structures, the city of Amman’s residential buildings were chosen as a case study. In this study, the common features of residential buildings in the city of Amman were identified and converted into parameters through which the initial design form, the number of floors, and the increase in the complexity of the configuration were determined, and then adding details by specifying the number of windows, adding them, adding doors, platforms, and staircases as a first step to achieve spatial congruence and sympathetically respond to context. The benefits of augmented reality were used as the following step to verify the design and adjust it to fit the surrounding physical context.

This approach was tested by 30 students from the University of Jordan, and the outputs were assessed using a questionnaire and a machine learning model. The findings indicate the effectiveness of the approach, and the students' feedback was overwhelmingly positive, according to the majority of participants 80% of the designs that resulted were in harmony with the surrounding context style, according to the findings of the examination using the machine learning model. This approach would aid efforts to achieve architectural harmony, particularly aesthetic harmony and continuity in the traditional area. The analysis and interpretation of the surrounding context’s architectural style through rules and procedural modeling aids in the generation of new patterns that have the characteristics of the architecture of residential buildings in Amman; thus, it is hoped that the findings of this study will serve as a valuable reference point for the innovative approach to contextual design.

Procedural modeling integrated with AR technology opens up new possibilities for architects, such as developing an application able to man-ipulate 3D elements and adjust design parameters inside the immersive environments to provide additional support.

There were some limitations that may have affected the study, the application does not allow the possibility of displaying texture and materials for buildings to give more realism. Also, it was difficult to select a larger number of students from the study sample due to the conditions of the Covid-19 epidemic.

## Declarations

### Author contribution statement

Maha AlFadalat: Conceived and designed the experiments; Performed the experiments; Analyzed and interpreted the data; Wrote the paper.

Wael Al-Azhari: Contributed reagents, materials, analysis tools or data; Wrote the paper.

### Funding statement

This research did not receive any specific grant from funding agencies in the public, commercial, or not-for-profit sectors.

### Data availability statement

Data included in article/supp. material/referenced in article.

### Declaration of interest’s statement

The authors declare no conflict of interest.

### Additional information

No additional information is available for this paper.
